# The Degradation of Airway Epithelial Tight Junctions in Asthma Under High Airway Pressure Is Probably Mediated by Piezo-1

**DOI:** 10.3389/fphys.2021.637790

**Published:** 2021-04-01

**Authors:** Jia Zhou, Xiang-dong Zhou, Rui Xu, Xian-zhi Du, Qi Li, Bin Li, Guo-yue Zhang, Ling-xiu Chen, Juliy M. Perelman, Victor P. Kolosov

**Affiliations:** ^1^Department of Respiratory Medicine, The First Affiliated Hospital of Chongqing Medical University, Chongqing, China; ^2^Department of Respiratory Medicine, The First Affiliated Hospital of Hainan Medical University, Haikou, China; ^3^Key Laboratory of Emergency and Trauma of Ministry of Education, Hainan Medical University, Haikou, China; ^4^Department of Respiratory Medicine, The Second Affiliated Hospital of Chongqing Medical University, Chongqing, China; ^5^Department of Respiratory and Critical Care Medicine, People’s Hospital of Fengjie, Chongqing, China; ^6^Department of Respiratory Medicine, Chongqing Three Gorges Central Hospital, Chongqing, China; ^7^Far Eastern Scientific Center of Physiology and Pathology of Respiration, Russian Academy of Medical Sciences, Blagoveshchensk, Russia

**Keywords:** asthma, small airway epithelium, auto-PEEP, tight junction, piezo-1

## Abstract

Full functioning of the airway physical barrier depends on cellular integrity, which is coordinated by a series of tight junction (TJ) proteins. Due to airway spasm, edema, and mucus obstruction, positive end-expiratory alveolar pressure (also termed auto-PEEP) is a common pathophysiological phenomenon, especially in acute asthma attack. However, the influence of auto-PEEP on small airway epithelial TJs is currently unclear. We performed studies to investigate the effect of extra pressure on small airway epithelial TJs and its mechanism. The results first confirmed that a novel mechanosensitive receptor, piezo-1, was highly expressed in the airway epithelium of asthmatic mice. Extra pressure induced the degradation of occludin, ZO-1 and claudin-18 in primary human small airway epithelial cells (HSAECs), resulting in a decrease in transepithelial electrical resistance (TER) and an increase in cell layer permeability. Through *in vitro* investigations, we observed that exogenous pressure stimulation could elevate the intracellular calcium concentration ([Ca^2+^]_*i*_) in HSAECs. Downregulation of piezo-1 with siRNA and pretreatment with BAPTA-AM or ALLN reduced the degradation of TJs and attenuated the impairment of TJ function induced by exogenous pressure. These findings indicate the critical role of piezo-1/[Ca^2+^]_*i*_/calpain signaling in the regulation of small airway TJs under extra pressure stimulation.

## Introduction

Asthma, one of the most common chronic respiratory inflammatory disease worldwide, is characterized by airway hyperresponsiveness and variable airflow obstruction, followed by airway epithelial repair and remodeling (Dhami et al., 2015). Due to its physical barrier function, the respiratory epithelium acts as the first protective defence against allergens, viruses, microorganisms and particulate matter. Full functioning of the airway physical barrier depends on cellular integrity with the expression and coordinate interaction of a series of proteins formed in cell-cell junctional complexes called tight junctions (TJs) (Godfrey, 1997). TJs are distributed at the subapical regions of airway epithelial cells, where they separate the apical domain from the basolateral domain and maintain cell polarity in the human airway epithelium. A TJ ‘belt’ in the airway epithelium selectively regulates the paracellular passage of intercellular substances and ions and restricts the lateral flow of material in adjacent cell membranes (Ganesan et al., 2013). In asthma, the degradation of TJ proteins and subsequent increase in airway epithelial cell permeability are involved in the process of airway hyperreactivity and exacerbation of asthma. These findings have been demonstrated by the collection of bronchial biopsy samples from asthma patients (Xiao et al., 2011).

Positive end-expiratory alveolar pressure resulting from dynamic hyperinflation due to airway spasm, oedema, and mucus obstruction in asthma has been termed intrinsic PEEP (also termed auto-PEEP). Auto-PEEP is a common pathophysiological phenomenon in acute asthma attack and leads to pathologically positive pressure on the terminal airways and alveoli (Broseghini et al., 1988; Busk et al., 2013). Although different measurement methods offer slightly different auto-PEEP values, it is generally believed that the auto-PEEP in patients with asthma attack is approximately 10 cmH_2_O. However, in some patients with severe asthma attack, the auto-PEEP can reach as high as approximately 20 cmH_2_O (Ranieri et al., 1993; Leatherman and Ravenscraft, 1996). However, in asthma attack, the influence of auto-PEEP on TJ barrier function of the terminal airway epithelium and its specific mechanisms are currently unclear. The Piezo channel is a novel mechanosensitive cation channel discovered in recent years with its family members Piezo-1 (Fam38a) and Piezo2 (Fam38b). First detected in murine neuroblastoma cells, Piezo-1 was recently identified to be expressed in the epithelium and vascular endothelium of different organs and tissues (Wang et al., 2016; Dalghi et al., 2019; Bai et al., 2020). The Piezo-1 channel on the cell membrane is activated by sensing the tension of the cell membrane and changes in the cell membrane curvature. Activated Piezo-1 channels allow the transmembrane distribution of cations, such as calcium, prompting the cellular response to tension stress (Gottlieb and Sachs, 2012). Current research on the distribution of the Piezo channel in the lungs suggests that Piezo-2 is distributed in airway sensory neurons, where it senses and perform an essential role in the Hering-Breuer reflex Nonomura et al., 2017). Unlike Piezo-2, Piezo-1 is widely expressed in the airway epithelium, including the alveolar epithelium (Wang et al., 2017; Zhong et al., 2018). Therefore, an *in vivo* study in a mouse model and *in vitro* cellular study were performed to investigate the role of the Piezo-1 channel in regulating the epithelial TJs of terminal small airway tracks under auto-PEEP in asthma attack.

## Materials and Methods

### Animals and Asthma Mouse Model

Sixteen adult female BALB/c mice, approximately 8 weeks of age, were purchased from the Laboratory Animal Center of Chongqing Medical University and maintained under specific pathogen-free conditions. The animal experiments were performed in accordance with the National Institutes of Health Guide for the Care and Use of Laboratory Animals (NIH publication No. 8023, revised 1978). Mice were randomly divided into 2 groups: the control group and the asthma group. Mice in the asthma group were sensitized by intraperitoneal injection of 0.2 ml of sensitizing agents (in PBS as a solvent) on days 0 and 7. The sensitizing agents were 25 μg of ovalbumin (OVA) (A5503-5G, grade V, Sigma-Aldrich, Pudong, Shanghai, China) and 1 mg of aluminium hydroxide. Mice in the control group were intraperitoneally injected with an equal volume of PBS. Following intraperitoneal injection of sensitizing agents, mice in the asthma group received 10 ml of aerosolized stimulant twice a day on days 21–28. The aerosolization agent was 1% OVA formulated in PBS (0.1 g of OVA + 10 ml of PBS). Mice in the control group were aerosolized with 10 ml of PBS twice a day on days 21–28. Mice in both the control group and asthma group were sacrificed 24 h after the last OVA challenge.

### Immunohistochemistry

The lungs were harvested from the mice and immediately fixed with 4% paraformaldehyde for 24 h. Then, the lung tissues were embedded in paraffin, cut into sections and prepared for immunohistochemistry according to the instructions of an SP-kit. After quenching endogenous peroxidase with 3% H_2_O_2_, tissue slides were incubated with 1% normal rabbit serum to minimize non-specific binding. Next, the slides were incubated with rabbit polyclonal anti-piezo1 antibody (1:200, Abcam, Pudong, Shanghai, China) at room temperature for 1 h in a dark, humidified box. After washing with PBS 3 times, slides were incubated with FITC-conjugated goat anti-rabbit IgG (1:100, ZSGB-BIO, Beijing, China) for 30 min. After washing with PBS, slides were then incubated with 100 ng/ml DAPI for 10 min. After washing with PBS 3 times, the slides were embedded in 50% glycerol. Tissues were visualized using a confocal microscope (TCS SP2, Leica, Germany). Representative images were obtained with a digital camera and then processed with ImageJ 1.5.

### Cell Culture and Extra Pressure Treatment

Primary human small airway epithelial cells (HSAECs, PCS-301-010) were purchased from the American Type Culture Collection (Manassas, VA, United States). Cells were cultured in Dulbecco’s modified Eagle’s medium (DMEM) with 10% FBS (Thermo Fisher Scientific, Inc., Waltham, MA, United States), penicillin (100 IU/ml), streptomycin (100 IU/ml), and bronchial epithelial cell growth agent (PCS-300040, ATCC, Manassas, VA, United States) at 37°C in a 5% CO_2_ incubator and passaged when the cells were 70-80% confluent. Before experimental grouping, cells were counted and transferred to microporous membrane (aperture of 0.4 μm) inserts at the air-liquid interface (ALI) in equal amounts. The upper chamber containing the microporous membrane was then inserted into a 24-well plate. Each 24-well plate well contained 600 μl of the culture medium mentioned above. Cells were maintained at 37°C in a 5% CO_2_ incubator, and the culture medium was changed every 2 days until a complete cell layer was formed. For further experiments, 24-well plates were separately placed in a homemade incubation chamber. A simple incubation chamber with a piston at the top for pressure regulation and a small pressure detection device was designed. The sides of the chamber were designed to contain pipes that connected a unidirectional valve for gas exchange. Cells were divided into 2 groups: the control (CTL) group and the pressure culture (PC) group. Cells in the CTL group were placed in the homemade incubation chamber without additional pressure on the piston above the chamber. Cells in the PC group were placed in the chamber with additional pressure on the piston above the chamber to maintain the pressure in the chamber at 10 cmH_2_O (A schematic diagram of the chamber is shown in Supplementary Figure 1). Both chambers containing cells in the CTL group or PC group were placed in an incubator containing 5% CO_2_ and 95% air.

### Small Interfering RNA (siRNA) Preparation and Transduction

The pGC-silencer-U6/Neo/GFP vector was purchased from Santa Cruz Biotechnology (Shanghai) Co., Ltd. (Pudong, Shanghai, China). siRNA against human piezo-1 and a control (CTL) siRNA were designed and chemically synthesized by Shanghai GeneChem Co., Ltd. (Shanghai, China). The piezo-1 siRNA sequence was 5′-GCCUCGUGGUCUACAAGAUTT-3′ (for the sense strand) and 5′-AUCUUGUAGACCACGAGGCTT-3′ (for the antisense strand). CTL siRNA was scrambled siRNA containing a GU content similar to that of piezo-1 siRNA. Prior to transfection, cells in exponential growth phase were plated in 6-well cell culture plates and incubated at 37°C for 12 h. Following 3 washes with PBS to avoid any interference caused by antibiotics or serum, the cells were transfected by FuGENE^®^ HD reagent (E2311, Promega Corporation, Madison, WI, United States) with piezo-1-specific siRNA or CTL siRNA (20 μg of DNA:60 μl of transfection reagent) at 22°C for 15 min according to the manufacturer’s protocol. Following transfection, cells were washed with PBS 3 times and incubated in complete culture medium for 24 h at 37°C prior to western blotting and subsequent experiments.

Female BALB/c mice were anesthetized intraperitoneally with midazolam before siRNA transfection *in vivo*. A fine catheter was placed into the nostril. Through the catheter, 60 μl RNAse-free water (containing 60 μg siRNA) were perfused at a rate of 6 μl/min using a micro syringe pump. Oxygen was supplied during the whole procedure in order to avoid hypoxia. The mice were perfused thrice (every second day). Seventy -two hours after the last transfection, bronchoalveolar lavage fluid (BALF) was collected, and centrifuged. The supernatant was prepared for IgM detection according to the instructions of mice IgM ELISA kits (Westang, Shanghai, China). The left lung was digested for western blotting.

### Evaluation of HSAEC Epithelial Barrier Function

Transepithelial electrical resistance (TER) was measured with a Millicell-ERS system (Millipore Co.) For measurements, the basal electrode was covered with equilibrated ALI medium. Then, 50 μL of ALI medium was added to cover the microporous membrane surface. The apical electrode was placed into the apical liquid. The measurements were performed immediately after positioning of the apical electrodes. The TER values (Ω × cm^2^) were calculated with the following equation: (TER_sample_ – TER_blank_) × surface area.

The paracellular permeability was measured using a fluorescein isothiocyanate (FITC)-dextran (4 kD, Sigma-Aldrich) permeation test. Briefly, the upper chamber containing a microporous membrane was washed with PBS 3 times. Phenol red (0.2 ml) and serum-free DMEM (containing 0.5 mg/L FITC-dextran) were added to the upper chamber. Phenol red (1.0 ml) and serum-free DMEM (containing 0.5 mg/L dextran without FITC conjunction) were added to the lower chamber. Then, the cells were incubated in a dark incubator with 5% CO_2_ and 95% air for 2 h. Liquid samples (0.1 ml and 0.5 ml) were collected from the upper and lower chambers, respectively, for fluorescence measurement. The excitation and emission wavelengths were 488 and 525 nm, respectively. The concentrations of FITC-dextran in the upper and lower chambers were calculated based on a fluorescence standard curve. The permeability coefficient of dextran (Pd) was calculated with the following formula: Pd(cm/s) = ([A]/T) × (1/A) × (V/[L]), where [A] is the concentration of FITC-dextran in the lower chamber, T is the time(s), A is the area of the bottom of the upper chamber (cm^2^), V is the volume of liquid in the lower chamber, and [L] is the concentration of FITC-dextran in the upper chamber.

### Measurement of Intracellular Ca^2+^ Concentration ([Ca^2+^]_*i*_)

The microporous membrane covered with HSAECs was cut from the upper chamber and placed into a 24-well plate. Each microporous membrane was loaded with 5 μM Fura 2-AM (Invitrogen, Carlsbad, CA, United States) Cells were then incubated in the dark for 1 h at 37°C and washed with PBS for 20 min to remove residual dye. Fluorescence was measured at excitation wavelengths of 340 and 380 nm and an emission wavelength of 510 nm. Data were acquired and are expressed as fluorescence ratios (F340/F380).

### Western Blot Analysis to Detect the Expression of Occludin, Zo-1, Claudin-18, and Piezo-1

The expression levels of proteins of interest in the cellular lysate supernatants were detected by western blotting. Generally, cells were washed with PBS 3 times and lysed on ice for 20 min with IP lysis buffer (P0013, Beyotime Institute of Biotechnology, Songjiang, Shanghai, China). The lysates were then centrifuged at 20,000 × *g* for 15 min at 4°C to remove nuclei and intact cells. Supernatants were standardized for equal protein concentration (5 μg/μl) using a bicinchoninic acid protein assay kit (Beyotime Institute of Biotechnology, Songjiang, Shanghai, China). Following separation by SDS-PAGE, proteins were transferred onto polyvinylidene difluoride (PVDF) membranes, which were blocked with 5% skimmed milk for 1 h at room temperature. PVDF membranes were then incubated with primary rabbit antibodies against ZO-1 (Abcam, ab96587) at a 1:1000 dilution, piezo-1 (Abcam, ab128245) at a 1:500 dilution, claudin-18 (Abcam, ab203563) at a 1:2000 dilution, occludin (Abcam, ab235986) at a 1:2000 dilution, or β-actin (ZSGB-BIO, Beijing China) at a 1:1000 dilution at 4°C overnight. The PVDF membranes were washed with TBST 3 times and subsequently incubated with horseradish peroxidase (HRP)-conjugated goat anti-rabbit IgG secondary antibody (ZSGB-BIO, Beijing China) at a 1:1000 dilution for 2 h. Blots were visualized using enhanced chemiluminescence following the manufacturer’s specifications (KeyGen, Nanjing, China). The intensity of each band was measured using a Fluor-S multi-imager and Quantity One software (Bio-Rad, Hercules, CA, United States). The expression levels of the proteins of interest were normalized to that of β-actin.

### Cell Immunochemistry to Detect TJ Proteins

The microporous membrane covered with HSAECs was cut from the upper chamber and placed into a 24-well plate. After 3 washes with PBS, the cells were fixed with 4% paraformaldehyde for 10 min and washed again with PBS. The fixed cells were permeabilized with 0.1% Triton X-100 in PBS for 10 min and then washed 3 times with PBS again. The cells were then blocked in 5% goat serum for 60 min and incubated with rabbit primary antibodies against ZO-1 (Abcam, ab96587) at a 1:200 dilution, claudin-18 (Abcam, ab203563) at a 1:200 dilution, and occludin (Abcam, ab235986) at a 1:100 dilution at 4°C overnight. After washing with PBS, the cells were incubated for 60 min with Alexa Fluor^®^ 594-conjugated goat anti-rabbit IgG (ZF-0516, ZSGB-BIO, Beijing China) at a 1:200 dilution in the dark. After washing with PBS, membranes were embedded in 50% glycerol. Cells were visualized using a confocal microscope (TCS SP2, Leica, Germany). Representative images were obtained with a digital camera and then processed with Adobe Photoshop CS2.

### Statistics

Normally distributed data are described using the mean and standard deviation (SD), and differences between two groups were analyzed using Student’s *t*-test. More than two groups were compared using one-way analysis of variance (ANOVA), followed by Bonferroni analysis. Non-normally distributed data are described using the median and interquartile range (IQR), and differences between groups were analysed using the Mann-Whitney test. All statistical analyses were performed using SPSS (Statistical Package for the Social Sciences) version 17 software (SPSS, Inc.). All statistical graphs were drawn using GraphPad Prism 8.

## Results

### Piezo-1 Might Be Responsible for the Alterations in the Increased Airway Permeability in Asthmatic Model Mice

**FIGURE 1:**
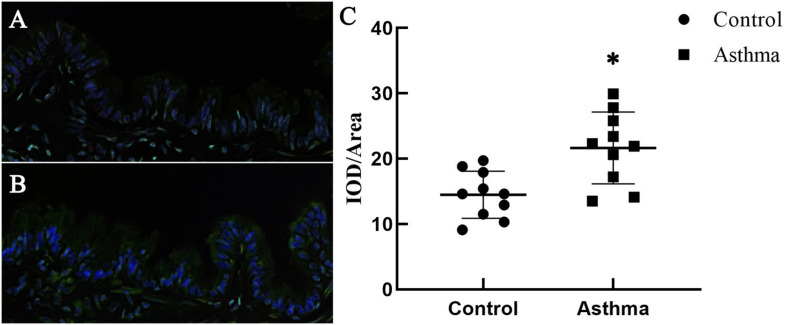
Expression of Piezo-1 in the bronchial epithelium. Mouse lung tissues were immunostained with rabbit anti-piezo1 antibody (1:200) at room temperature in a humidified environment for 1 h and then incubated with FITC-linked goat anti-rabbit antibody (1:100, ZSGB-BIO, Beijing, China) for 30 min. Cell nuclei were visualized by staining with DAPI. The IOD/area of Piezo-1 (green fluorescence) in the bronchial epithelium was recorded under a confocal microscope. **(A)** A representative photo of normal mice is shown. **(B)** A representative photo of asthmatic mice is shown. **(C)** Scatter diagram of Piezo-1 protein expression in the airway epithelium (IOD/area). ^∗^*P* < 0.05 vs. control mice.

**FIGURE 2:**
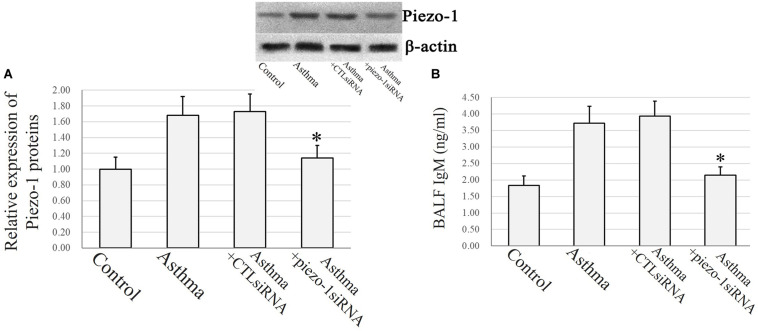
Piezo-1 might be responsible for the increased airway permeability in asthmatic mice model. **(A)** Western blot assay to detect the piezo-1 protein. Tissue piezo-1 levels are described as relative expression normalized to that of β-actin. Data are represented as the mean ± SD, *n* = 6. *P < 0.05 vs. asthmatic mice. **(B)** Airway permeability based on the transit of IgM to the airway was increased in asthmatic mice. Piezo-1siRNA airway transduction partially abolished the increased IgM. ^∗^*P* < 0.05 vs. asthmatic mice. Data are represented as mean ± SD, *n* = 6.

Immunochemistry and confocal microscopy were applied to visualize Piezo-1 in bronchial epithelial cells from model mice. Piezo-1 was expressed in the bronchial epithelium of normal and asthmatic mice. The Piezo-1 levels found in the immunohistochemical experiment in the bronchial epithelium are expressed as the integrated optical density/area (IOD/area). The IOD/area for Piezo-1 in the bronchial epithelium was slightly higher in asthmatic mice than in normal control mice (21.6 ± 5.5 vs. 14.5 ± 3.6; *P* < 0.05) (Figure 1). To determine the effect of siRNA transfection in lung by airway perfusion, lung tissue was digested for western blot detecting for piezo-1. We found a reduction in the piezo-1 protein content to about 32.1% 72 h after transfection with piezo-1siRNA (Figure 2A). Later, we examined the degree of airway permeability using the detection of large protein IgM (Frampton et al., 1999) in bronchoalveolar lavage fluid (BALF). The level of IgM in the BALF was markedly elevated in the asthmatic mice compared to the normal control mice. Piezo-1siRNA airway transduction partially abolished the increased IgM in the BALF in asthmatic mice (Figure 2B).

### MTT Assay to Assess Cell Viability

**FIGURE 3:**
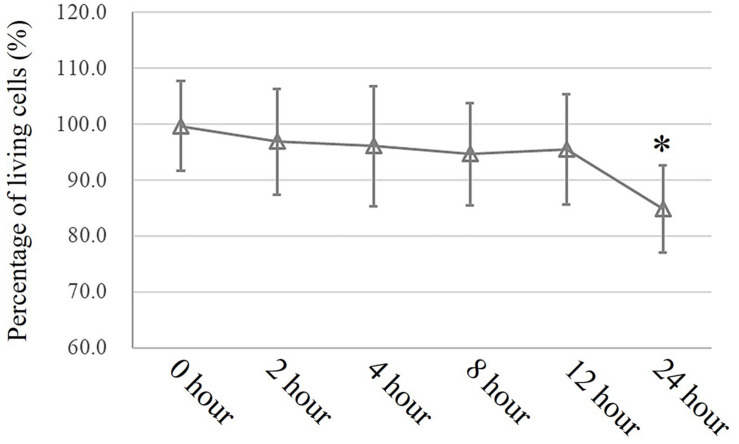
MTT assay to assess cell viability. Cells propagated under normal atmospheric pressure were set as controls. Data are represented as the mean ± SD, *n* = 6, ^∗^*P* < 0.05 vs. 0 h exposure in HSEACs.

In an *in vitro* study, a conventional MTT assay was performed to evaluate cell viability after exposure to pressure for different durations. According to the MTT assay, an extra 10 cmH_2_O of pressure for 16 h caused a significant reduction in HSAEC cell viability. Thus, 12 h was selected as the appropriate exposure time for the following experiments (Figure 3).

### TJs Were Disrupted in Small Airway Epithelial Cells *in vitro* Under Extra Pressure

**FIGURE 4:**
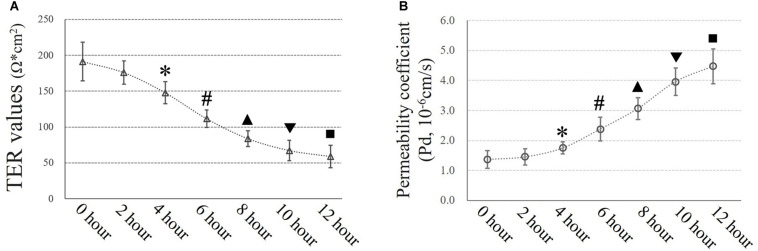
Time-dependent high-pressure assay to assess TJ function. **(A)** TER values of HSAECs. ^∗^*P* < 0.05, ^#^*P* < 0.05, ^▲^*P* < 0.05, ^▼^*P* < 0.05, ^■^*P* < 0.05 vs. the initial (0 h) TER level, respectively. **(B)** Paracellular permeability assay for HSAECs. The results are described as permeability coefficients (Pd values). ^∗^*P* < 0.05, ^#^*P* < 0.05, ^▲^*P* < 0.05, ^▼^*P* < 0.05, ^■^*P* < 0.05 vs. the initial (0 h) Pd level, respectively.

Airway epithelial function was estimated by TER (Ω × cm^2^) and FITC-dextran permeability tests as described in the experimental section. Exposure to 10 cmH_2_O of additional pressure caused a time-dependent reduction in TER in HSAECs. The TER value began to decrease significantly after 4 h of exposure to additional pressure. In the first 4 h, the TER value decreased to 77.3% of the initial level. Meanwhile, the Pd increased by 27.7% of the initial level. At 12 h of exposure, the TER value had decreased to approximately 30.9% of the initial level, and the Pd had increased to 3.3-fold the original value (Figure 4).

### Extra Pressure Increased the Intracellular Ca^2+^ Concentration ([Ca^2+^]_*i*_) by Activating Piezo-1 in HSAECs

**FIGURE 5:**
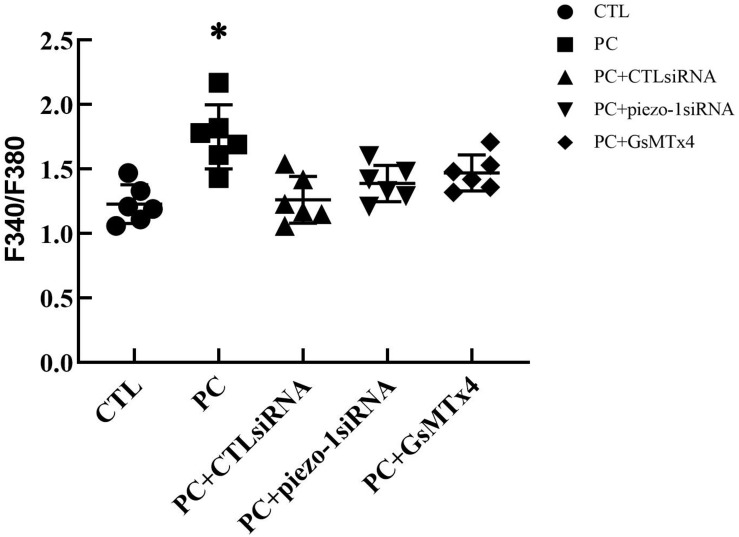
Analysis of [Ca^2+^]_*i*_ in HSAECs. Cells propagated under normal atmospheric pressure were set as controls (CTL, the baseline). HSAECs exposed to 10 cmH_2_O of pressure for 12 h were marked as the ‘pressure culture’ (PC) group. Data are expressed in a scatter diagram. Bars represent the mean and SD. Differences between groups were analysed using one-way ANOVA, followed by Bonferroni’s test. ^∗^*P* < 0.05 vs. ‘PC + piezo-1 siRNA’ or ‘PC + GsMTx4.’

The additional 10 cmH_2_O of pressure evoked an increase in [Ca^2+^]_*i*_ in HSAECs. Following exposure to high-pressure culture for 12 h, the F340/F380 ratio increased from 1.23 ± 0.15 at baseline to 1.75 ± 0.25 *(P* < 0.05 vs. baseline). Pretreatment with the piezo-1 inhibitor GsMTx4 partially attenuated the increase in F340/F380 after high-pressure culture. The F340/F380 ratio increased to 1.47 ± 0.14 in GsMTx4-pretreated cells. To further demonstrate the crucial role of piezo-1 in pressure-induced calcium influx in HSAECs, we constructed piezo-1-knockdown (piezo-1 KD) HSAECs with piezo-1-specific siRNA transfection (as mentioned in the ‘Materials and Methods’ section). The efficiency of piezo-1 downregulation was verified by western blotting, as shown in the ‘Supplementary Materials’ section (Supplementary Figure 2). In piezo-1-specific siRNA-transfected cells, the F340/F380 value increased to 1.39 ± 0.14 after culture under extra pressure (Figure 5). These results indicate that extra pressure triggers a piezo-1-dependent increase in [Ca^2+^]_*i*_ manner.

### Extra Pressure Induced the Degradation of Occludin, ZO-1, and Claudin-18 in the Small Airway Epithelium

**FIGURE 6:**
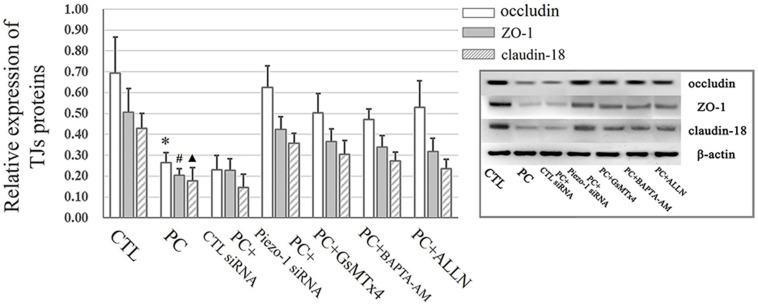
Western blot assay to detect TJ proteins. Cells propagated under normal atmospheric pressure were set as the control (CTL). HSAECs exposed to 10 cmH_2_O of pressure for 12 h were marked as the ‘pressure culture’ (PC) group. Data are represented as the mean ± SD, *n* = 6. Differences between groups were analysed using one-way ANOVA, followed by Bonferroni’s test. ^∗^*P* < 0.05 vs. the occludin level in ‘PC + piezo-1 siRNA,’ ‘PC + GsMTx4,’ ‘PC + BAPTA-AM,’ or ‘PC + ALLN.’ ^#^*P* < 0.05 vs. the ZO-1 level in ‘PC + piezo-1 siRNA,’ ‘PC + GsMTx4,’ ‘PC + BAPTA-AM,’ or ‘PC + ALLN.’ ^▲^*P* < 0.05 vs. the claudin-18 level in ‘PC + piezo-1 siRNA,’ ‘PC + GsMTx4,’ ‘PC + BAPTA-AM,’ or ‘PC + ALLN.’

**FIGURE 7:**
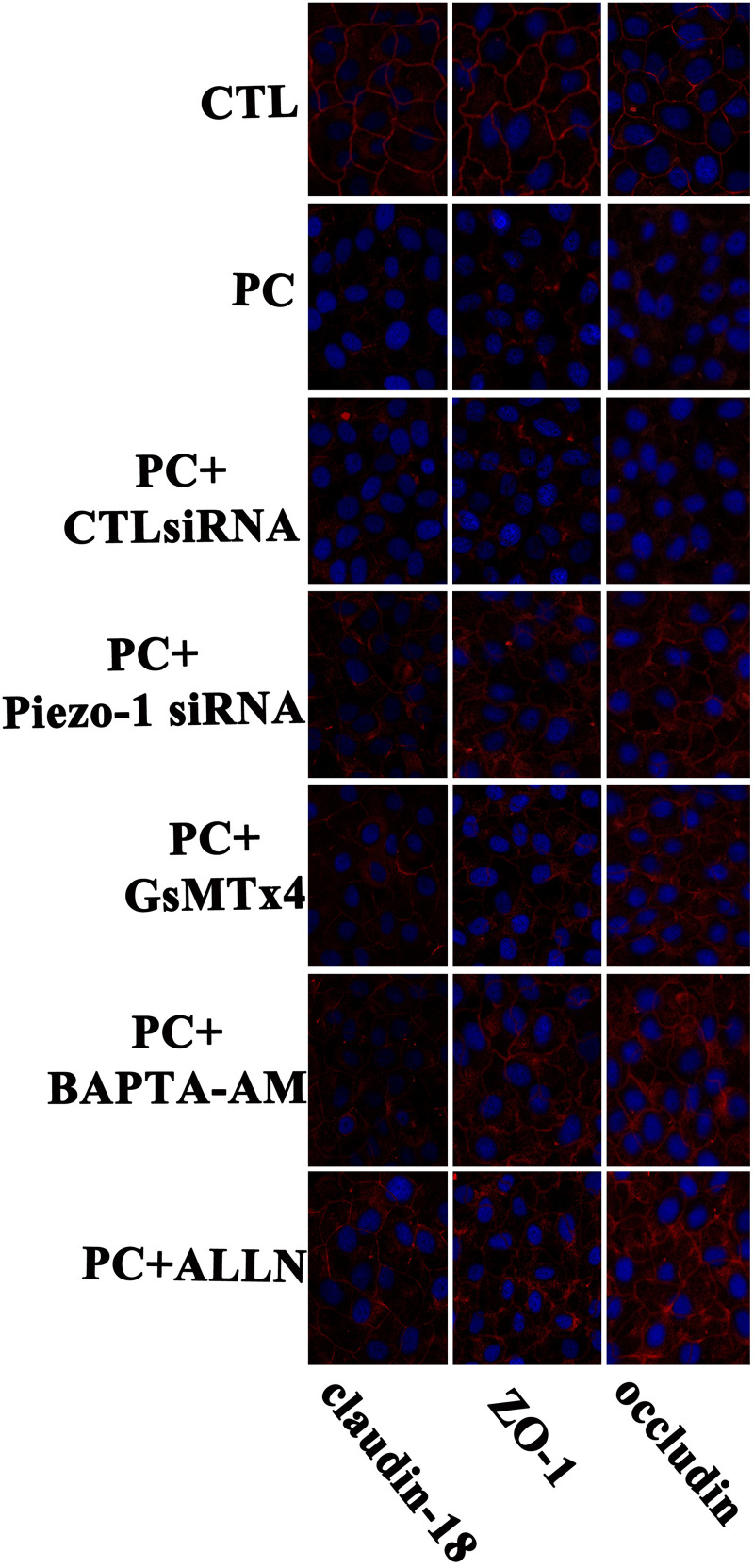
Immunofluorescent staining for of TJ proteins (occludin, ZO-1 and claudin-18) in HSAECs. Cells propagated under normal atmospheric pressure were set as the control (CTL). Cells exposed to 10 cmH_2_O of pressure for 12 h were marked as the ‘pressure culture’ (PC) group. TJ proteins were visualized by Alexa Fluor^®^ 594-linked secondary antibody (red fluorescence). Nuclei were labelled with DAPI (blue fluorescence). The results shown are representative photos of the outcomes obtained in 6 independent experiments.

Occludin and ZO-1 were detected as they play an indispensable role in regulating barrier tightness and a critical role in the establishment of airway belt-like TJs, respectively (Furuse, 2010; Matter and Balda, 2014). Claudin-18, a lung-specific claudin that is highly associated with airway epithelial barrier dysfunction in asthma, was also detected (Sweerus et al., 2017; Yang et al., 2019). To further assess whether the disruption in occludin, ZO-1, and claudin-18 occurred because of additional pressure exposure, we first investigated cellular occludin, ZO-1, and claudin-18 by western blot assay. The results indicated that an additional 10 cmH_2_O of pressure promoted the degradation of occludin, ZO-1, and claudin-18 in HSAECs (Figure 6, vertical panels 1 and 2, ‘CTL’ vs. ‘PC’). To visualize the distribution of TJ proteins in epithelial HSEAC cells, cellular immunofluorescence and confocal microscopy were performed. High-pressure exposure decreased intracellular TJs and caused discontinuous occludin, ZO-1, and claudin-18 staining in HSAECs (Figure 7, ‘CTL’ vs. ‘PC’). These findings were consistent with our western blot findings.

### The Degradation of Occludin, ZO-1, and Claudin-18 Under Extra Pressure Stimulation Might Rely on Activation of the Piezo-1 Channel and Subsequent Calcium/Calpain Cascade Activation

According to the results of western blot assessment, pretreatment with GsMTx4, a piezo-1 inhibitor, or Piezo-1 siRNA transfection partially attenuated the degradation of occludin, ZO-1 and claudin-18 (Figure 6). As visualized by cellular immunochemistry, GsMTx4 treatment or Piezo-1-specific siRNA transfection attenuated intracellular occludin, ZO-1 or claudin-18 degradation and partially preserved the continuity of intercellular TJ bands (Figure 7). These results indicated a crucial role of Piezo-1 in the degradation of small airway TJ proteins under additional pressure stimulation. Since Ca^2+^ is an important second messenger with crucial roles in the regulation of TJs and because activated piezo-1 allows Ca^2+^ influx (Gangwar et al., 2017; Du et al., 2020), we detected the influence of additional pressure on TJs following pretreatment with the Ca^2+^ chelator BAPTA-AM (10 μM). Similarly, pretreatment with the Ca^2+^ chelator partially reduced the pressure-induced degradation of occludin, ZO-1, and claudin-18 (Figure 6) and preserved the continuity of TJ bands in HSAECs (Figure 7). As calpain, a calcium-dependent protease, has been demonstrated to play an essential role in cytoskeletal degradation and tight junction impairment (Wang et al., 2012; Alluri et al., 2016), we inhibited calpain protease activity with ALLN (30 μM). ALLN partially abolished the degradation of occludin, ZO-1, and claudin-18 induced by additional pressure (Figure 6). As visualized by cellular immunochemistry, HSAECs pretreated with ALLN retained several TJ bands under high-pressure exposure, which indicated that calpain also participated in the degradation of occludin, ZO-1, and claudin-18 (Figure 7).

### The Piezo-1/[Ca^2+^]_*i*_/Calpain Cascade Is Highly Associated With the Disruption of Small Airway Epithelial Cells *in vitro* Under Extra Pressure

**FIGURE 8:**
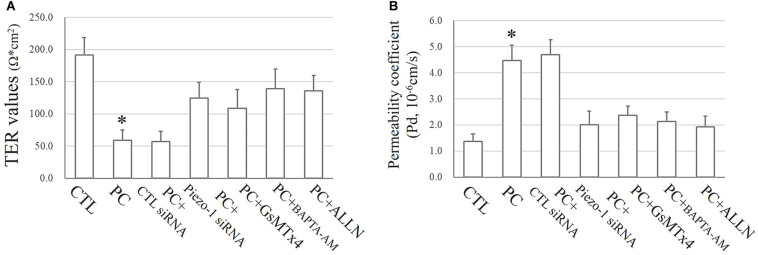
Epithelial TJ function assessments. Cells propagating normal atmospheric pressure were set as controls (CTLs). HSAECs exposed to a 10 cmH_2_O pressure for 12 h were marked as the ‘pressure cultured (PC)’ group. Data are represented as the mean ± SD, *n* = 6. Differences between groups were analysed using one-way ANOVA, followed by Bonferroni’s test. **(A)** TER values of HSAECs. ^∗^*P* < 0.05 vs. ‘PC + piezo-1 siRNA,’ ‘PC + GsMTx4,’ ‘PC + BAPTA-AM,’ and ‘PC + ALLN.’ **(B)** Paracellular permeability assay in HSAECs. The results are described as permeability coefficients (Pd 587 values). ^∗^*P* < 0.05 vs. ‘PC + piezo-1 siRNA,’ ‘PC + GsMTx4,’ ‘PC + BAPTA-AM,’ or ‘PC + ALLN.’

As mentioned above, the TJ function in HSAECs was estimated by TER and FITC-dextran permeability tests. Exposure to extra pressure for 12 h caused a reduction in TER (59.1 ± 15.7 Ω⋅cm^2^) as mentioned above. Pretreatment with GsMTx4 or piezo-1 siRNA to inhibit activation of the piezo-1 channel attenuated the reduction in TER by extra-pressure exposure. The TER of HSAECs pretreated with the Ca^2+^ chelator BAPTA-AM decreased to 139.1 ± 30.3 Ω⋅cm^2^, which was significantly higher than that of untreated cells exposed to extra pressure. Pretreatment with the calpain protease inhibitor ALLN also partially reduced the decrease in TER in HSAECs under high-pressure exposure (Figure 8A). A further FITC-dextran permeability test was performed to estimate paracellular permeability. The results were highly consistent with the TER outcomes (Figure 8B). These results strongly indicate that a mechanism involving Piezo-1/[Ca^2+^]_*i*_/calpain participates in the disruption of small airway epithelial cells *in vitro* under extra pressure.

## Discussion

The airway epithelium constitutes the first cell layer that prevents harmful substances, such as bacteria, viruses, and some physical and chemical pollutants, from reaching the lungs (Ganesan et al., 2013; Denney and Ho, 2018). The full barrier function of the airway epithelium directly depends on the integrity of TJs. In airway tracks, TJs composed mainly of heteromeric occludin, zonula occluden and claudin protein complexes form a sealed interface between adjacent airway epithelial cells. Damage to TJ proteins is one of the initiating factors of impaired airway epithelial barrier function. It also occurs in the majority of inflammatory respiratory diseases, such as asthma, COPD, pneumonia and cystic fibrosis (Tam et al., 2011; Castellani et al., 2017; Aghapour et al., 2018; Gon and Hashimoto, 2018). Existing studies, including those in bronchial mucosal biopsies from asthmatic patients (Xiao et al., 2011), have confirmed the downregulation of TJ protein expression and the defective functioning of epithelial TJs. Furthermore, increased airway epithelial permeability caused by impaired TJ function may lead to an increased risk of infection and the persistence of airway hyperresponsiveness (Kicic et al., 2016; Gon and Hashimoto, 2018).

In patients with acute exacerbation of asthma, iPEEP is a common pathophysiological phenomenon due to broncho-spasm and excessive dynamic hyperinflation of the lungs. In this study, we selected primary human small airway epithelial cells (HSAECs) for the *in vitro* experiment mainly because lesions in the small airway are typical of patients with asthma attack. An air-liquid external culture with 10 cmH_2_O of extra pressure was adopted to better simulate the small airway environment in asthmatic patients *in vitro* (Ranieri et al., 1993; Leatherman and Ravenscraft, 1996). Our study found that 10 cmH_2_O of extra pressure for 4 h sufficiently induces a defect in TJ function and an increase in permeability of the small airway epithelial cell layer. In a study of ventilator-associated lung injury in rats, both low tidal volume and high tidal volume ventilation resulted in the degradation of occluding (Liu et al., 2014). Although this study focused on TJs in the entire lung tissues and our study was restricted to the small airway epithelium, both studies report similar conclusions on extra pressure-induced TJ deficiency.

Mechanical transduction is the process by which cells respond and adapt to external stimuli. In this process, mechanical force stimuli need to be converted into electrical or chemical signals. Piezo is a mechanosensitive transmembrane channel that responds to mechanical stress over a wide and dynamic range of external mechanical stimuli (Ge et al., 2015).Piezo-1 and piezo-2 have already been demonstrated in lung tissue. According to previous studies, piezo-1 is widely expressed in the bronchus and alveolar and vascular endothelial cells (Gudipaty et al., 2017; Wang et al., 2017). However, until now, most reported effects of piezo-2 have been in the neural reflex arc, such as gentle touch sensation mediated by the Merkel cell-neurite complex (Wu et al., 2017) and the Hering-Breuer reflex in the lung (Nonomura et al., 2017). Hence, in our *in vivo* and *in vitro* experiments, we focused on the piezo-1 channel. According to our *in vivo* data, expression of the piezo-1 receptor in bronchial epithelial cells was slightly higher in asthmatic mice than in normal mice. Interestingly, our result coincides with the results of a previous study in a bladder partial outflow tract obstruction model in rats (Michishita et al., 2016). In this completed study, researchers found that 7 days after rat bladder outlet obstruction, the piezo-1 expression level was increased in whole-bladder tissues and expected that this increase in expression might be involved in a compensatory mechanism (Michishita et al., 2016).

Our research data indicate that extra pressure induces a strong reduction in the occludin and ZO-1 proteins in the small airway epithelial cell layer and the redistribution of occludin and ZO-1 in intercellular TJ belts. Although the pathogenesis of asthma is considered diverse, impairment of airway epithelial function and disordered TJ protein expression may represent one common pathological features of asthma (Cereijido et al., 1988; Holgate, 2009). Earlier studies in bronchial epithelium biopsies from asthmatic patients suggested the significant downregulation of ZO-1 and occludin in the epithelium of asthmatic patients (Boskabady and Snashall, 1992; Xiao et al., 2011). Several studies have suggested that pro-inflammatory cytokines, such as IL-13 and IL-4, which are involved in the pathogenesis of asthma, contribute to the degradation of ZO-1 and occluding (Ahdieh et al., 2001; Xiao et al., 2011). Other studies indicated the impairment of ZO-1 and the apical junctional complex by cigarette smoking (Shaykhiev et al., 2011; Tatsuta et al., 2019). Our observations extend these findings from another perspective. We focused on high expiratory pressure (iPEEP), a common pathophysiological phenomenon in asthma, and demonstrated the relationship between the degradation of TJs and iPEEP. Furthermore, we found that claudin-18, a lung-specific claudin, was significantly decreased and redistributed following exposure to extra pressure. Previous studies, both *in vivo* and *in vitro*, have suggested that claudin-18 plays a vital role in resisting exogenous aeroantigens (Sweerus et al., 2017; Lee et al., 2020). The consequences of claudin-18 deficiency are highly related to airway hyperreaction and delayed airway epithelial repair (Sweerus et al., 2017). Therefore, based on the conclusions of our study, we infer that impaired claudin-18 in the terminal small airway due to extra pressure in consistent asthma attack is not conducive to asthma relief.

In this study, we demonstrated a [Ca^2+^]_*i*_- and calpain-dependent mechanism of airway TJ degradation. Under normal cellular physiological conditions, calpain retains relatively low enzyme activity and plays a critical role in regulating cytoskeleton renewal and vesicle transport and maintaining normal cell metabolism (Kampfl et al., 1997; Chakraborti et al., 2012; Fei et al., 2013; Ma, 2013). However, under intracellular calcium overload, calpain can be completely activated, resulting in a series of effects, such as cell damage, cytoskeleton degradation, and intercellular TJ impairment. These effects have already been confirmed in studies of clpain enzyme-induced vascular endothelial damage induced by inflammatory factors (Hannah et al., 2011).

## Conclusion

In conclusion, our study demonstrates that the novel mechanosensitive receptor piezo-1 mediates impairment of small airway epithelial function der extra pressure. Our results further support the involvement of [Ca^2+^]_*i*_/calpain-associated mechanisms. Furthermore, these conclusions might explain the cause of defective small airway epithelial function, that is, the state of excess terminal airway iPEEP in bronchial asthma. From the perspective of impaired epithelial function, these conclusions may provide a new strategy for the control and treatment of bronchial asthma.

## Data Availability Statement

The original contributions presented in the study are included in the article/Supplementary Material, further inquiries can be directed to the corresponding author/s.

## Ethics Statement

The animal study was reviewed and approved by Biomedical Research Ethics Committee of Chongqing Medical University.

## Author Contributions

RX designed the research, participated in the statistics, and study coordination. JZ carried out Western blot studies and participated in drafting the manuscript. XD conducted the confocal microscopy analysis. XZ and LC conducted the asthma mouse model and performed the *in vivo* studies. QL and JP performed ALI cell culture and cell transfection. VK finished the [Ca^2+^]_*i*_ concentration measurement. GZ performed TER analyse and the paracellular permeating test. BL performed animal experiments and collected the BALF. All authors contributed to the article and approved the submitted version.

## Conflict of Interest

The authors declare that the research was conducted in the absence of any commercial or financial relationships that could be construed as a potential conflict of interest.

## Supplementary Material

Supplementary Figure 1 The schematic diagram of the pressurized cell culture chamber. **(A)** An air pressure detection device; **(B)** Cell culture plate; **(C)** The sealed piston; **(D)** A tube connecting to the cell incubator; **(E)** A controllable valve.

Supplementary Figure 2 Western blot assay to detect the piezo-1 protein. Cells were propagated under normal atmospheric pressure. HSAECs were transfected with control (CTL) siRNA or piezo-1 siRNA. Piezo-1 levels are described as relative expression normalized to that of β-actin. Data are represented as the mean ± SD, *n* = 6. ^∗^*P* < 0.05 vs. wild-type (WT) cell.
