# Supplementary material for: The Degradation of Airway Epithelial Tight Junctions in Asthma Under High Airway Pressure Is Probably Mediated by Piezo-1
Source: Front Physiol. 2021 Apr 1;12:637790. doi: 10.3389/fphys.2021.637790 (PMC8047413; doi:10.3389/fphys.2021.637790)
Supplement: Supplementary Figure 1 — The schematic diagram of the pressurized cell culture chamber. (A) An air pressure detection device; (B) Cell culture plate; (C) The sealed piston; (D) A tube connecting to the cell incubator; (E) A controllable valve. [file Table_1.docx]

Supplementary Material

# Supplementary Figures


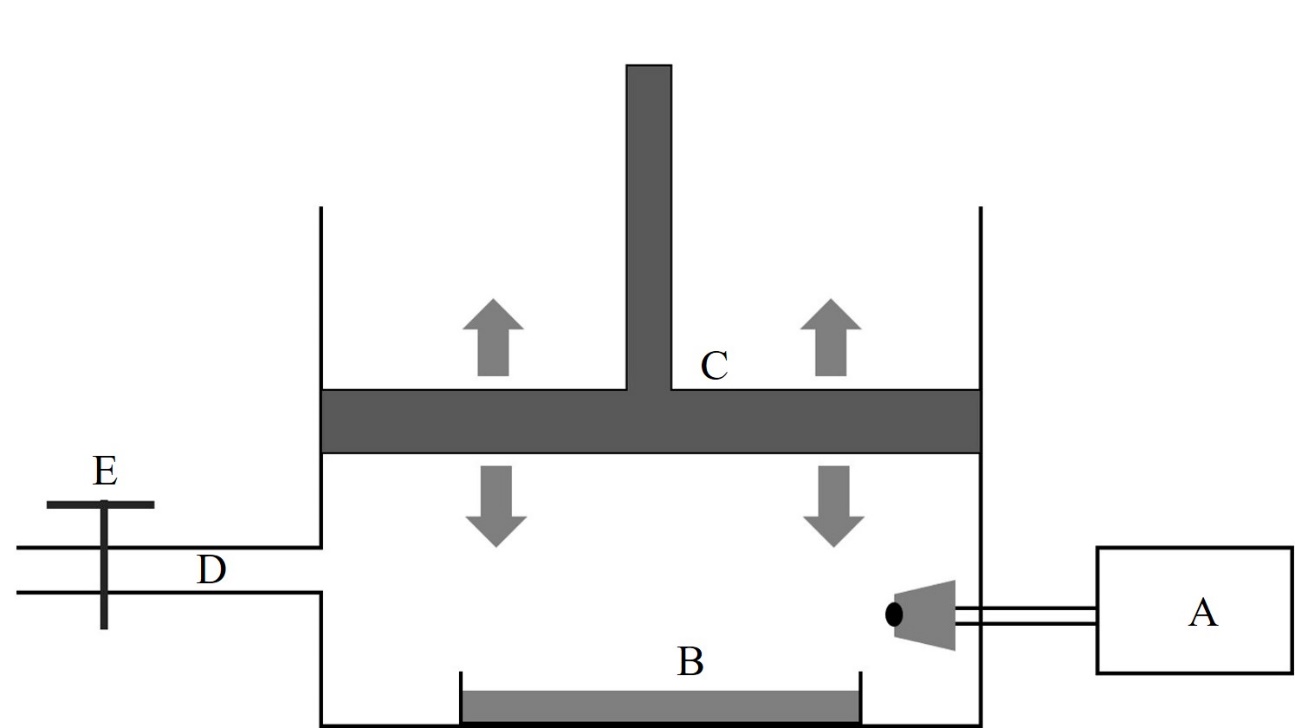


**Figure S1. The schematic diagram of the pressurized cell culture chamber. A.** An air pressure detection device; **B.** Cell culture plate; **C.** The sealed piston; **D.** A tube connecting to the cell incubator; **E.** A controllable valve.


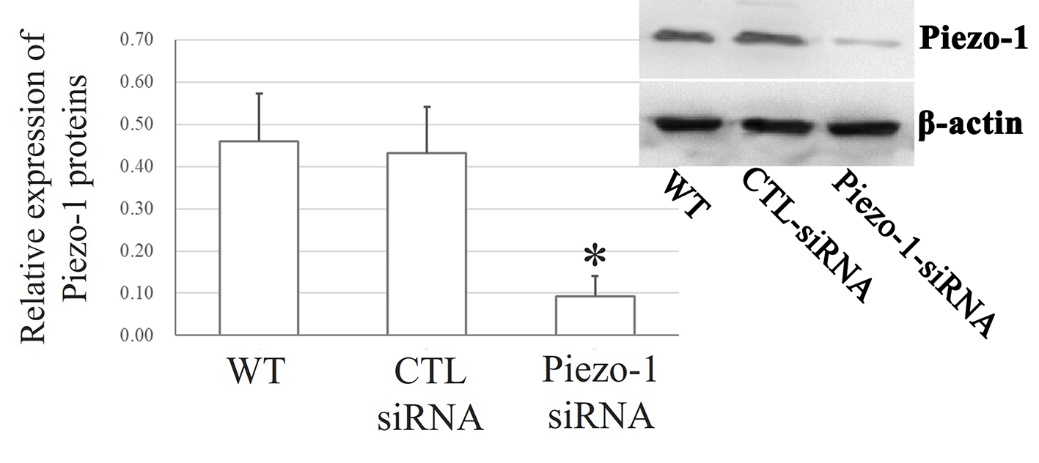


**Figure S2. Western blot assay to detect the piezo-1 protein.** Cells were propagated under normal atmospheric pressure. HSAECs were transfected with control (CTL) siRNA or piezo-1 siRNA. Piezo-1 levels are described as relative expression normalized to that of β-actin. Data are represented as the mean±SD, n=6. **P<*0.05 vs. wild-type (WT) cell..
